# Use of oral moist tobacco (snus) in puberty and its association with asthma in the population-based RHINESSA study

**DOI:** 10.1136/bmjresp-2024-002401

**Published:** 2024-07-22

**Authors:** Juan Pablo López-Cervantes, Vivi Schlünssen, Chamara Senaratna, Simone Accordini, Francisco Javier Callejas, Karl A Franklin, Mathias Holm, Nils Oskar Jogi, Andrei Malinovschi, Anna Oudin, Torben Sigsgaard, Elin Helga Thorarinsdottir, Christer Janson, Randi Jacobsen Bertelsen, Cecilie Svanes

**Affiliations:** 1 Department of Global Public Health and Primary Care, Centre for International Health, University of Bergen Faculty of Medicine and Dentistry, Bergen, Norway; 2 Department of Occupational Medicine, Haukeland University Hospital, Bergen, Norway; 3 Department of Public Health, Research Unit for Environment, Occupation and Health, Danish Ramazzini Centre, Aarhus University, Aarhus, Denmark; 4 Allergy and Lung Health Unit, Centre for Epidemiology and Biostatistics, School of Population and Global Health, University of Melbourne VCCC, Victoria, Melbourne, Australia; 5 Unit of Epidemiology and Medical Statistics, Department of Diagnostics and Public Health, University of Verona, Verona, Italy; 6 Department of Respiratory Medicine, Albacete University Hospital Complex, Albacete, Spain; 7 Department of Surgical and Perioperative Sciences, Surgery, Umeå University, Umea, Sweden; 8 Department of Occupational and Environmental Medicine, Sahlgrenska University Hospital, Goteborg, Sweden; 9 Department of Medical Sciences, Clinical Physiology, Uppsala University, Uppsala, Sweden; 10 Department of Public Health and Clinical Medicine, Umeå University, Umea, Sweden; 11 Primary Health Care of the Capital Area, Reykjavik, Iceland; 12 Faculty of Medicine, University of Iceland, Reykjavik, Iceland; 13 Department of Medical Sciences: Respiratory, Allergy and Sleep Research, Uppsala University, Uppsala, Sweden; 14 Department of Clinical Science, University of Bergen, Bergen, Norway

**Keywords:** Tobacco and the lung, Asthma, Asthma Epidemiology, Surveys and Questionnaires

## Abstract

**Objectives:**

To investigate the association of early snus use initiation (≤15 years of age) with asthma and asthma symptoms.

**Design:**

Cross-sectional analysis of a population-based cohort.

**Setting:**

Study centres in Norway, Sweden, Iceland, Denmark and Estonia, from 2016 to 2019.

**Participants:**

9002 male and female participants above 15 years of age of the Respiratory Health in Northern Europe, Spain and Australia study.

**Main outcome measures:**

Current asthma and asthma symptoms.

**Results:**

The median age of study participants was 28 years (range 15–53) and 58% were women. 20% had used snus, 29% men and 14% women. Overall, 26% of males and 14% of females using snus started ≤15 years of age. Early snus use initiation was associated with having three or more asthma symptoms (OR 2.70; 95% CI 1.46 to 5.00) and a higher asthma symptom score (β-coefficient (β) 0.35; 95% CI 0.07 to 0.63) in women. These associations were weak in men (OR 1.23; 95% CI 0.78 to 1.94; β 0.16; 95% CI −0.06 to 0.38, respectively). There was evidence for an association of early snus initiation with current asthma (OR 1.72; 95% CI 0.88 to 3.37 in women; OR 1.31; 95% CI 0.84 to 2.06 in men). A sensitivity analysis among participants without smoking history showed stronger estimates for all three outcomes, in both men and women, statistically significant for three or more asthma symptoms in women (OR 3.28; 95% CI 1.18 to 9.10). Finally, no consistent associations with asthma outcomes were found for starting snus after age 15 years.

**Conclusions:**

Snus initiation in puberty was associated with higher likelihood of asthma and asthma symptoms, with the highest estimates in females and those without smoking history. These results raise concerns about the health adversities of early snus initiation and emphasise the need for public health initiatives to protect young people from this tobacco product.

**WHAT IS ALREADY KNOWN ON THIS TOPIC**

- Limited evidence suggests that people who use snus may have higher risk of asthma and chronic bronchitis.
- Tobacco smoking starting in puberty seems more harmful for both the user and their future offspring, but health effects of early initiation of snus or other tobacco products remain unknown.

**WHAT THIS STUDY ADDS**

- Snus use starting before or at the age of 15 years was associated with more asthma symptoms, particularly in women.
- The associations of early snus use with asthma showed consistent, even stronger estimates in participants who have never smoked.
- Puberty appears to be a vulnerable period also for use of a nicotine-containing tobacco products such as snus.

**HOW THIS STUDY MIGHT AFFECT RESEARCH, PRACTICE OR POLICY**

- This information can inform efforts to prevent the use of snus in young people and potentially reduce the burden of asthma in the future.

## Introduction

 Oral moist tobacco, commonly known as snus, is a tobacco product with high nicotine content, typically provided in small sachets or as powder.^1^ Over recent years, it has been increasingly used by young individuals in several countries, especially in Northern Europe, where the prevalence of use among those younger than 25 years has reached 15%–20%, including women.^2 3^ The increasing popularity of snus is often attributed to the perception that it is a less harmful alternative to tobacco smoking and is presumed to be a possible, though controversial, means of smoking cessation.^4^ However, the current evidence suggests that the adverse health effects associated with snus use should not be underestimated and warrant further investigation.^4^,^7^

The literature on snus and health outcomes is very limited. Respiratory health effects of snus were investigated in a large Swedish cross-sectional study, which revealed that snus use among people without smoking history was associated with increased risk of asthma, asthma-related symptoms and chronic bronchitis.^6^ Snus use has otherwise been linked to an increased risk of both all-cause and cardiovascular mortality among people who currently use snus as compared with those who have never used tobacco products in a pooled analysis of prospective cohort studies^5^; and to higher risk of ischaemic stroke in another prospective cohort study.^7^

Nicotine, polyaromatic hydrocarbons, tobacco-specific nitrosamines, pesticides and heavy metals are among the most common substances in snus.^1^ An average person who uses snus may keep the snus pouches or powder in the mouth for several hours per day implying prolonged contact between several chemical substances in snus and the oral mucosa.^8 9^ Nicotine in snus is absorbed through the oral mucosa into the systemic circulation and further binds to the nicotine acetylcholine receptors expressed by human bronchial and endothelial cells. After binding, nicotine exerts direct and indirect effects linked to lung, cardiovascular and brain changes.^10^,^12^ This is believed to be a biologically plausible mechanism for disease development related to snus use.

During stages of organ development and growth, the lungs are considered particularly more vulnerable to external factors.^13^ While susceptible exposure windows are known for tobacco smoking, exploration in the context of snus use is still lacking.^14 15^ Notably, the period before age 16 years is considered the most susceptible exposure window regarding smoking.^14^ Early initiation of smoking also appears to influence health of future offspring.^16^,^18^ Exposure effects often exhibit sex-specific patterns, attributed in part to the sex differences in maturation processes.^19 20^ We hypothesise that the age of initiation of snus use, like tobacco smoking, might be related to asthma risk in adulthood, following a sex-specific pattern. This study aims to investigate whether early initiation of snus use (≤15 years of age) is associated with asthma and asthma symptoms while considering potential modifications by sex.

## Materials and methods

### Study design, population and sample

For this study, we cross-sectionally analysed the data from the multicentre population-based Respiratory Health in Northern Europe, Spain and Australia study cohort (RHINESSA), carried out from 2013 to 2016. The cohort includes study participants of at least 3 generations from 10 centres: Norway (Bergen), Denmark (Aarhus), Sweden (Gothenburg, Uppsala and Umea), Iceland (Reykjavik), Estonia (Tartu), Spain (Albacete and Huelva) and Australia (Melbourne). These are the offspring of the participants of two population-based cohorts: the European Community Respiratory Heath Survey (ECRHS; www.ecrsh.org) and the Respiratory Health In Northern Europe (https://rhine.w.uib.no/) (detailed information elsewhere^21^).

**Figure 1:**
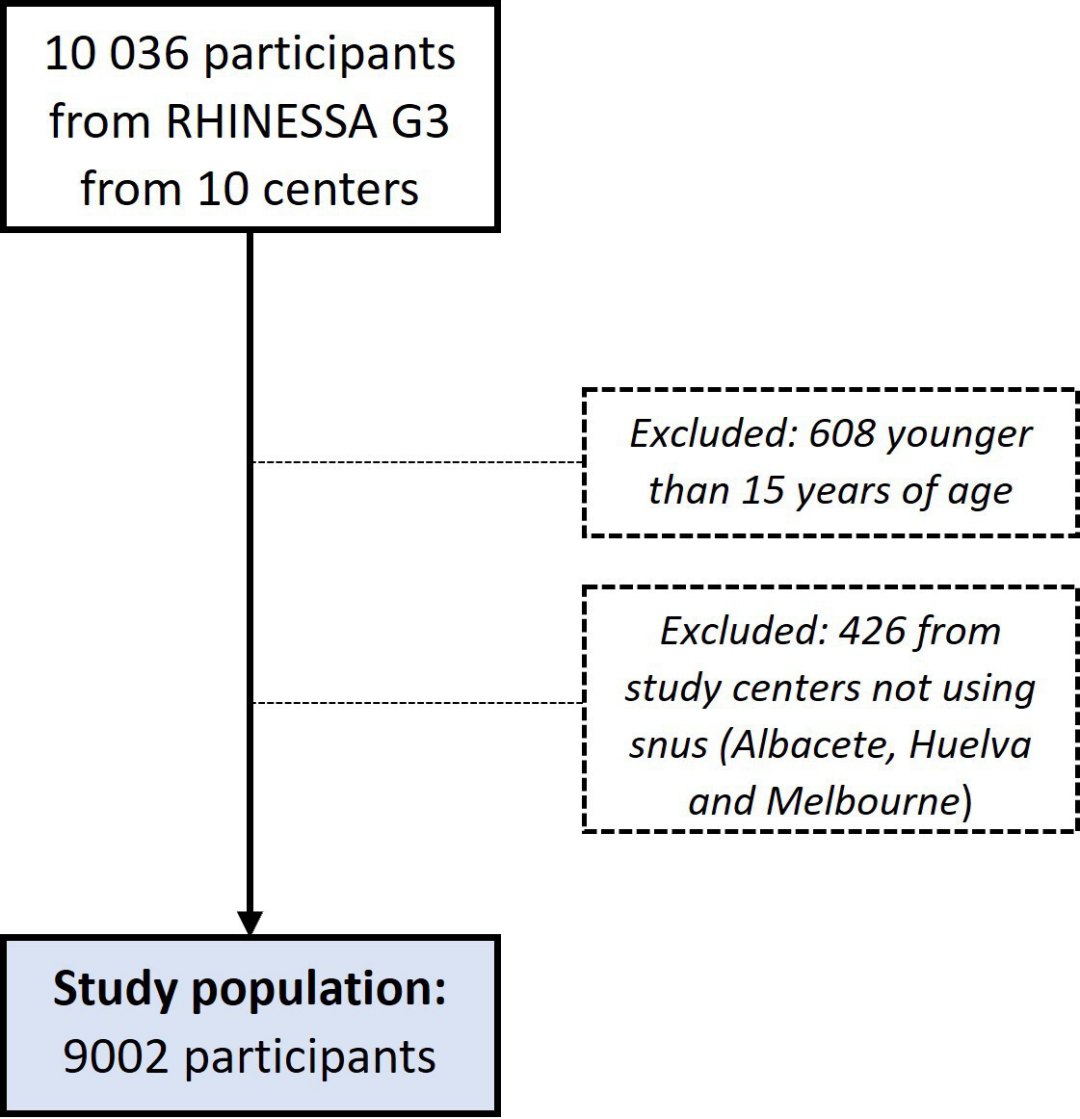
Flow chart of the selection of the study population. RHINESSA, Respiratory Health in Northern Europe, Spain and Australia.

Data were obtained from 10 036 participants from the ten centres that participated in the baseline RHINESSA study (RHINESSA third generation). Information about the history of consumption of tobacco-containing products was obtained using detailed questionnaires. Eligible participants were those older than 15 years of age with information on tobacco-consumption habits. We excluded 608 participants who were younger than 15 years and 426 participants from three centres (Albacete and Huelva in Spain and Melbourne in Australia) with no information about snus. Thus, the analyses included 9002 participants, as shown in figure 1.www.rhinessa.net

#### Exposures: age of starting snus

The following questions were used to define snus exposure: (a) do/did you use snus? If ‘yes’ to previous question: (b) age when starting using snus and (c) age when stopped using snus. These questions were used to define snus use and the age (in years) of initiation, setting a cut-off of 15 years of age to define early and late initiation of snus use. The categories for age of starting snus were generated as follows:

1. Never used snus (‘no’ to a).
2. Use of snus starting before or at age 15 years (‘yes’ to a and ‘≤15 years’ to b).
3. Use of snus starting after age 15 years (‘yes’ to a and ‘>15 years’ to b).

The age cut-off of 15 years was selected to capture snus exposure before reaching full puberty. This age was chosen because it aligns with the stage of normal organ development experienced by both girls and boys, a period that has been proposed as highly sensitive for the lungs. During this phase, several exposures, including tobacco, could influence respiratory health.^13 14^

#### Outcomes: current asthma and asthma symptoms

Participants provided detailed information about their own respiratory health, by responding to the standard questions developed for the ECRHS questionnaire. We defined current asthma as having asthma attacks and/or taking asthma medications within the last 12 months. Asthma symptoms were based on the eight questions included in Pekkanen *et al* asthma score (also based on the ECRHS questionnaire): wheeze with breathlessness in the last 12 months, wheeze without cold in the last 12 months, woken by tightness in chest in the last 12 months, woken by attack of shortness of breath in the last 12 months, woken by night cough in the last 12 months, ever had asthma, asthma attack in the last 12 months and currently taking asthma medication. The asthma symptom score was calculated as the sum of positive responses to these eight asthma symptoms, ranging from 0 to 8. This score was further categorised as <3 or ≥3 asthma symptoms.^22^

We additionally categorised asthma based on the presence or absence of allergic symptoms. As such, asthma with hay fever was defined as either using current asthma medicine or having asthma attacks or having three or more asthma symptoms and confirming a history of hay fever.

### Covariates

We used Directed Acyclic Graphs (DAG) to identify potential confounders and effect modifiers (online supplemental figure S1) in the association between age of starting snus use and current asthma and asthma symptoms. The DAG included all the covariates that were considered potentially important for the models and that were supported by scientific evidence.

Time of starting tobacco smoking was defined by using the following questions: (1) Are you a smoker? (2) Are you an ex-smoker? If yes to (1) or (2): (3) Age when started smoking; and if yes to (2): (4) Age when stopped smoking. The time of smoking initiation was categorised as below:

1. Never tobacco smoking (‘no’ to 1 and 2).
2. Tobacco smoking starting before or at age 15 years (‘yes’ to 1 and/or 2 and ‘≤15 years’ to 3).
3. Tobacco smoking starting after age 15 years (‘yes’ to 1 and/or 2 and ‘>15 years’ to 3).

Tobacco pack-years were determined by considering the daily cigarette consumption (as per the question ‘How many cigarettes per day?’) and the number of years smoking (as per the question ‘How many years have you smoked?’). Alternatively, the calculation could be based on the age at which participants began and ceased smoking (questions 3 and 4 above).

### Statistical analyses

The characteristics of the study population were described by sex and age of starting snus. The data were clustered by study centre and families, therefore, we analysed the associations between age of starting snus with current asthma and three or more asthma symptoms using multivariable multilevel (three levels and random intercept)^23^ logistic regression models. As for the analyses of the association of age of starting snus with the asthma symptom score (count), a negative binomial regression model was used given the overdispersed distribution of the data with the mean (0.98) being lower than the variance (2.33).^24^ We addressed missing values by only including participants with complete information on snus-consumption habits, without carrying out imputation of data. Only 184 persons out of 9002 were excluded from the regression models because they did not give information on age of onset of snus use (random loss of data). We made adjustments, as illustrated in the DAG in online supplemental figure S1, by considering age, the age when tobacco smoking started and the pack-years of tobacco smoking. The two latter were included given the plausible influence of the debut of tobacco smoking and intensity of smoking on the initiation of snus use at earlier or later age, as some participants may transition from smoking to using snus. Moreover, as tobacco smoking is widely recognised to be associated with respiratory symptoms, these factors were also taken into account.

In addition, we explored the potential interaction between sex and the age of starting snus use in relation to asthma and asthma symptoms. All models were stratified by sex for a comprehensive analysis. We conducted sensitivity analyses among participants who have never smoked and among those with asthma starting after age 15 years (no early-onset asthma—before age 15 years). By performing these sensitivity analyses, we excluded those participants with well-known risk factors for our outcomes.

Furthermore, we conducted an additional analysis to estimate the association of age of starting snus use and asthma with hay fever. Lastly, we investigated the association, considering reverse causality, between having experienced early-onset asthma (further categorised as having had asthma at ages ≤10 years and 11–15 years) and reporting use of tobacco products (categorised as ever snus use and ever tobacco smoking).

We considered an association to be significant if the estimates in the adjusted models had a conventional p≤0.05. For the inclusion of an interaction term or stratification, a less conservative value of ≤0.1 was employed as a cut-off. The statistical analyses were performed by using Stata V.17 (StataCorp).

### Patient and public involvement

Patients and the public were involved in several stages of the baseline RHINESSA study on which this analysis is based: patients were involved through a representative of the patient organisation, the Norwegian Asthma and Allergy Foundation and the public was represented through a user representative from the study participants and through stakeholder organisations; all representatives were invited to the annual meetings of the RHINESSA study. For more information, please refer to the official RHINESSA website (www.rhinessa.net) and in Svanes *et al*.^21^

## Results

### Main characteristics of the study sample (demographics and tobacco-consumption habits)

The general characteristics of the 9002 participants by sex and age of starting snus can be seen in table 1. Overall, women represented 58% of the sample and 1839 (20%) of the participants reported ever having used snus. The median age at participation was 28.3 years (range 15–53 years) and the majority of participants had normal BMI, attained college/university education and reported that they were engaged in frequent physical activities.

**Table 1:** Characteristics of the study population by sex and age of starting snus

	All (n=9002)	Men (n=3813; 42.5%)	Women (n=5189; 57.5%)	Never snus (n=7163)	Snus ≤15 years (n=392)	Snus >15 years (n=1417)
Centre						
Aarhus (DK)	1473	612 (42)	861 (58)	1414 (96)	8 (0.5)	49 (3.3)
Bergen (NO)	2046	875 (43)	1171 (57)	1501 (74)	92 (4.5)	447 (22)
Gothenburg (SE)	946	446 (47)	500 (53)	715 (76)	53 (5.6)	174 (18.4)
Umea (SE)	1306	561 (43)	745 (57)	861 (66)	130 (10.0)	309 (24)
Uppsala (SE)	1309	572 (44)	737 (56)	1003 (77)	76 (5.8)	223 (17.1)
Reykjavik (IS)	1247	466 (37)	781 (63)	1052 (84)	21 (1.7)	171 (13.7)
Tartu (EE)	675	281 (42)	394 (58)	617 (91)	12 (1.8)	44 (6.5)
Age (median, range)	28.3 (15–53)	28.3 (15–52)	28.3 (15–53)	28.2 (15–53)	28.7 (15–53)	28.9 (16–52)
Education						
Primary	434 (4.9)	200 (5.4)	234 (4.6)	377 (5.0)	16 (4.0)	39 (2.8)
Secondary	3595 (41)	1732 (47)	1863 (37)	2768 (40)	225 (58)	592 (42)
College/university	4771 (54)	1767 (48)	3004 (59)	3829 (55)	145 (38)	780 (55)
Exercise frequency						
Never	514 (6.2)	263 (7.5)	251 (5.0)	390 (6.0)	32 (8.5)	90 (6.5)
Sporadic	2672 (32)	1099 (31)	1573 (33)	2062 (32)	151 (40)	449 (32)
Frequent	5143 (62)	2125 (61)	3018 (62)	4083 (62)	192 (51)	852 (61)
Height (median, range)	173 (107–206)	182 (107–206)	168 (136–200)	172 (120–206)	178 (153–204)	176 (107–204)
BMI						
<18.5	370 (4.1)	91 (2.4)	279 (5.4)	318 (4.4)	8 (2.0)	42 (3.0)
≥18.5–24.9	5513 (61)	2109 (55)	3404 (66)	4474 (62)	206 (53)	817 (58)
≥25–29.9	2239 (25)	1222 (32)	1017 (20)	1681 (23)	116 (30)	433 (30)
≥30	880 (9.8)	391 (10.3)	489 (9.4)	690 (9.6)	62 (16.0)	125 (9.0)
Snus use						
Never	7163 (80)	2691 (71)	4472 (86)	–	–	–
Ever	1839 (20)	1122 (29)	717 (14)	–	–	–
Age of starting snus*						
≤15 years	392 (22)	292 (26)	100 (14)	–	–	–
>15 years	1417(78)	811 (74)	606 (86)	–	–	–
Tobacco smoking variables						
Never	5641 (67)	2395 (67)	3246 (66)	4638 (76)	164 (43)	632 (45)
≤15 years	1193 (14.0)	426 (12.0)	767 (15.7)	584 (9.6)	144 (38)	276 (20)
>15 years	1613 (19.0)	729 (21)	884 (18.1)	863 (14.2)	74 (19.0)	497 (35)
Packs/years (mean, SD)	4.52 (6.81)	5.06 (7.58)	4.13 (6.19)	4.98 (7.26)	2.72 (4.92)	4.0 (6.10)
Asthma outcomes						
Current asthma†	844 (9.5)	284 (7.6)	560 (10.9)	675 (9.5)	47 (12.0)	121 (8.6)
≥3 asthma symptoms‡	1099 (12.5)	359 (9.7)	740 (14.5)	844 (12.1)	63 (16.2)	190 (15.5)

Information missing for (by sex/age of starting snus): education (n=202/231); exercise frequency (n=673/701); age of starting snus (n=30); age of starting tobacco smoking (n=555/1008); packs/years of tobacco smoking-among ever smokers (n=155); asthma (never/ever) (n=52); current asthma medicine/attacks (n=28); ≥3 asthma symptoms (n=184).

Values are presented as (n, %) unless otherwise stated.

* Among ever snus users (data are available for n=1839).

†T Defined as: uThe use of asthma medication or attack of asthma in the last 12 months.

‡ Defined as:≥3 positive answers to eight8 questions, based on a modified version of the definition provided by Pekkanen *et al*^22^ , 2005: Wheeze with breathlessness in the last 12 months; wheeze without cold in the last 12 months; woken by tightness in chest in the last 12 months; woken by attack of shortness of breath in the last 12 months; woken by night cough in the last 12 months; ever had asthma; asthma attack in the last 12 months; currently taking asthma medication.

BMIbody mass indexDKDenmarkEEEstoniaISIslandNONorwaySESwedenyoyears old

**Figure 2:**
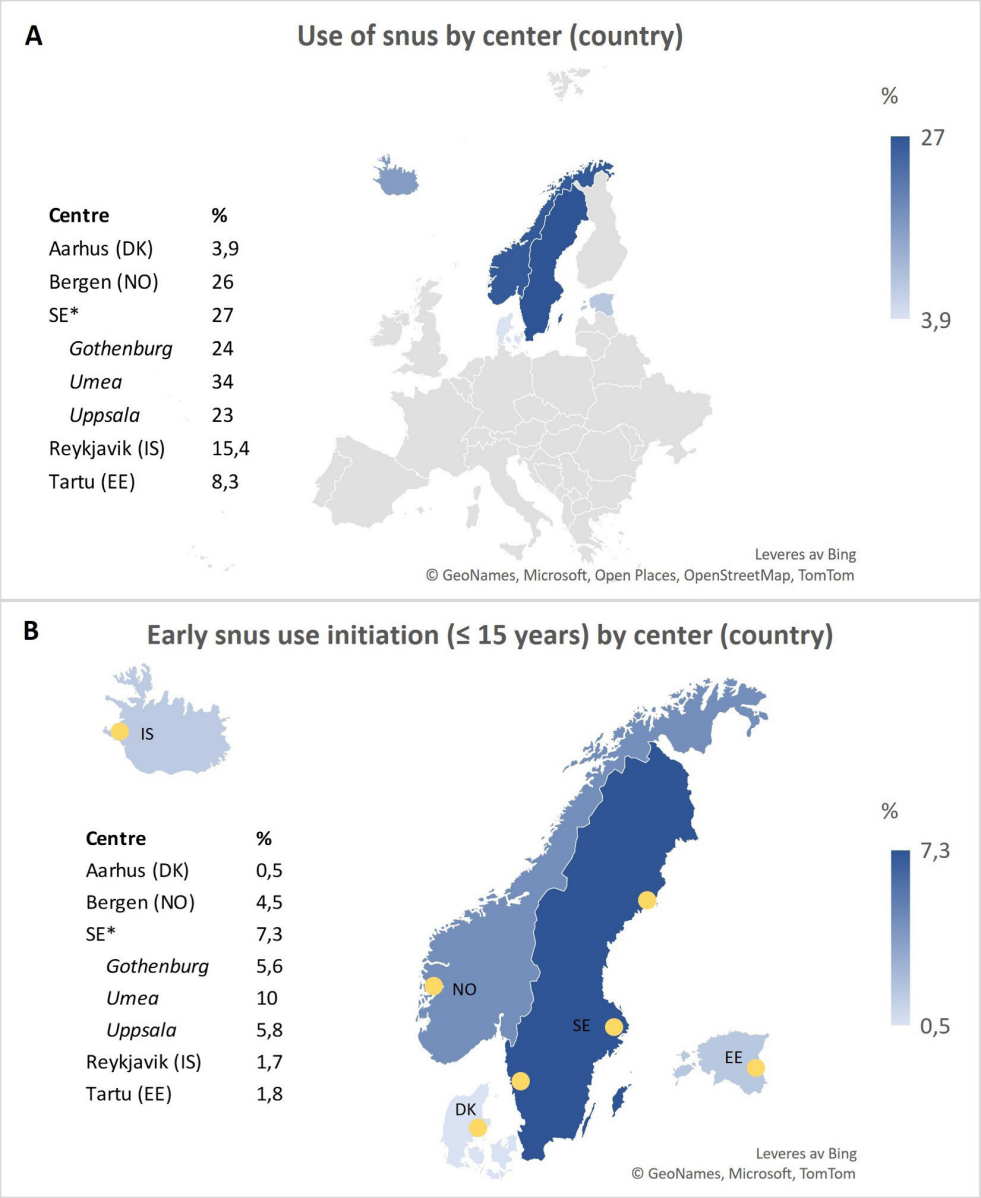
Maps showing the prevalence of snus use (**A**) and early snus use initiation (**B**) by study centre. *Average of three centres is shown for Sweden (SE). DK, Denmark; EE, Estonia; IS, Iceland; NO, Norway.

Snus use was most common in the three Swedish centres (Gothenburg, Umea and Uppsala) and in Bergen in Norway (24%, 34%, 23% and 26%, respectively) (figure 2A). The same study centres had the highest prevalence of starting snus ≤15 years of age, ranging from 4.5% to 10.0% (figure 2B). Men were more likely to use snus (29%) and to start using snus early (26%) as compared with women. One-third of the cohort reported ever smoking, with a similar proportion in men and women. With regard to asthma outcomes, both current asthma and asthma symptoms were more commonly reported in women from all study centres, except for Tartu, Estonia, where asthma was more prevalent in men. Participants with increasing BMI showed a trend of reporting more asthma, with this trend being more noticeable in women than in men (online supplemental table S1).

### Association between age of starting snus use and asthma outcomes

**Figure 3:**
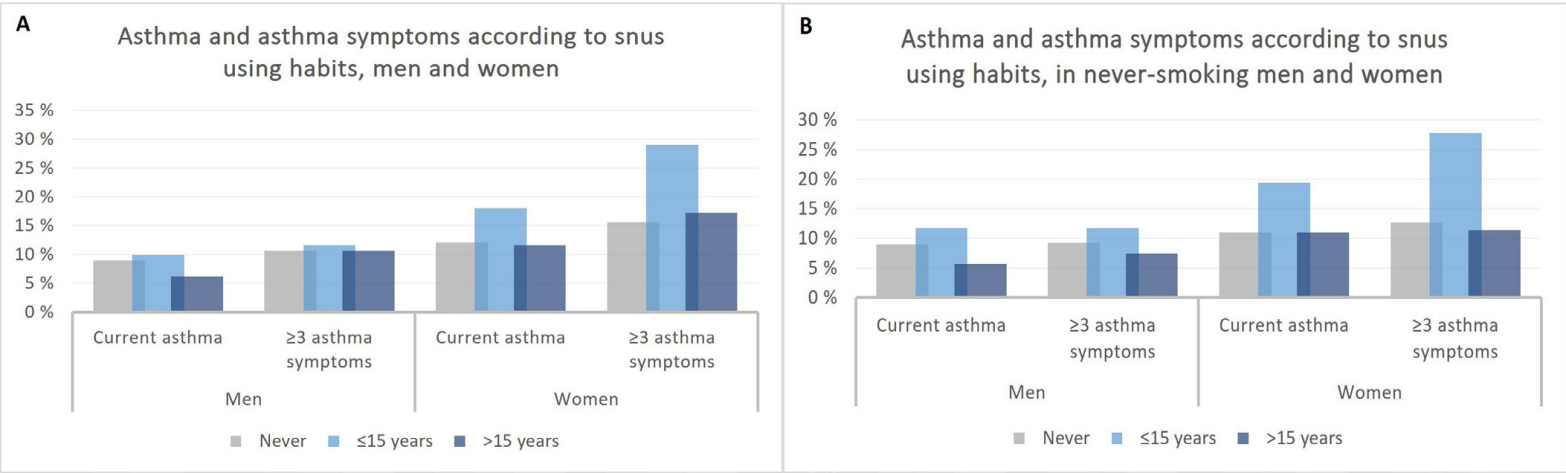
Graphs of the prevalence (%) of current asthma and asthma symptoms according to snus use habits by sex. Among all participants (**A**) and among those without smoking history (**B**). Data are available for (A) never: n=2262 (men) and n=3866 (women); ≤15 years: n=292 (men) and n=100 (women); >15 years: n=811 (men) and n=606 (women). Data are available for (B) never: n=1782 (men) and n=2856 (women); ≤15 years: n=128 (men) and n=36 (women); >15 years: n=386 (men) and n=246 (women).

The prevalence of current asthma and three or more asthma symptoms according to snus habits is presented in figure 3 for men and women. In female participants, those who had initiated snus use early had the highest prevalence of both current asthma (18.0%) and three or more asthma symptoms (29%) (figure 3A). In male participants, only a weak pattern was indicated. When restricted to participants who reported never smoking, higher prevalence of asthma outcomes in those starting snus use early was suggested in both men and women, most pronounced in women (figure 3B).

The adjusted models showed consistent positive associations between early snus use initiation with all three asthma outcomes, evident in both men and women. However, the associations were strongest in women (table 2). In female participants, the risk of having three or more asthma symptoms was almost tripled (adjusted OR 2.70; 95% CI 1.46 to 5.00) and the asthma symptom score was 0.35 units higher (adjusted β-coefficient (β) 0.35; 95% CI 0.07 to 0.63). The estimates for the same outcomes were only indicated in male participants (OR 1.23; 95% CI 0.78 to 1.94; β 0.16; 95% CI −0.06 to 0.38, respectively). The association between early snus use initiation and reporting three or more asthma symptoms was statistically significantly stronger in women than in men (p value for interaction between sex and early snus use initiation=0.023). Evidence of associations between early snus use and current asthma was indicated in both sexes (OR 1.72; 95% CI 0.88 to 3.37 in women; OR 1.31; 95% CI 0.84 to 2.06 in men). Having started using snus after 15 years of age was not consistently associated with asthma outcomes (table 2). Among participants without smoking history, the results were generally consistent, but the observed associations showed somewhat higher estimates in women (table 2).

**Table 2:** Asthma outcomes related to age of starting snus use in all the participants and in those without smoking history, by sex

All participants§	Current asthma*		≥3 asthma symptoms†		Asthma symptom score‡	
	Women	Men	Women	Men	Women	Men
Age of starting snus	OR (95% CI)		OR (95% CI)		β (95% CI)	
Never	1 (ref)	1 (ref)	1	1	0	0
≤15 years	1.72 (0.88 to 3.37)	1.31 (0.84 to 2.06)	2.70 (1.46 to 5.00)	1.23 (0.78 to 1.94)	0.35 (0.07 to 0.63)	0.16 (–0.06 to 0.38)
>15 years	1.00 (0.71 to 1.42)	0.85 (0.61 to 1.20)	1.20 (0.88 to 1.64)	1.08 (0.79 to 1.47)	0.16 (0.03 to 0.30)	−0.05 (–0.20 to 0.10)

No smoking history¶						
Age of starting snus						
Never	1 (ref)	1 (ref)	1	1	0	0
≤15 years	1.95 (0.70 to 5.44)	1.34 (0.73 to 2.44)	3.28 (1.18 to 9.10)	1.31 (0.70 to 2.45)	0.35 (–0.13 to 0.83)	0.31 (–0.02 to 0.63)
>15 years	0.95 (0.58 to 1.55)	0.62 (0.38 to 1.01)	0.88 (0.52 to 1.46)	0.75 (0.48 to 1.18)	0.10 (–0.11 to 0.31)	−0.14 (–0.35 to 0.08)

* Defined as: uThe use of asthma medication in the last 12 months and/or asthma attacks in the last 12 months. Data are available for n=8922 (all); n=5601 (no smoking history).

† Defined as:≥3 positive answers to eight 8 questions, based on a modified version of the definition provided by Pekkanen *et al*^22^ , 2005: Wheeze with breathlessness in the last 12 months; wheeze without cold in the last 12 months; woken by tightness in chest in the last 12 months; woken by attack of shortness of breath in the last 12 months; woken by night cough in the last 12 months; ever had asthma; asthma attack in the last 12 months; currently taking asthma medication. Data are available for n=8922 (all); n=5601 (no smoking history).

‡ Continuous scale based on the eight questions of the definition by Pekkanen *et al*, 2005. Data are available for n=8818 (all); n=5542 (no smoking history).

§ Adjusted for: age, age of starting tobacco smoking (never, ≤15 years old, >15 years old) and packs/years of tobacco smoking.

¶ Adjusted for age.

### Sensitivity analyses and test for reverse causality

When excluding participants with early-onset asthma, the outcomes remained crudely consistent, but the sex difference was less clear (online supplemental table S2). We further analysed the associations of snus use with two different phenotypes of asthma, ‘allergic asthma’ is defined as asthma with hay fever, and ‘non-allergic asthma’ is defined as asthma without hay fever. The results were consistent for both asthma phenotypes, with increased OR for both allergic and non-allergic asthma as associated with early snus use initiation among women (online supplemental table S3).

Finally, analyses on possible reverse causality for early-onset asthma with the ever use of snus and/or smoking tobacco showed no clear tendency for participants to report neither higher nor lower use of these tobacco-containing products (online supplemental table S4).

## Discussion

### Statement of principal findings

We investigated whether the age of starting the use of snus, a nicotine-containing tobacco product, was associated with asthma and asthma symptoms, in a cross-sectional analysis in 9002 participants of a large multinational population-based cohort. We found that starting snus use before or at age 15 years was associated with more asthma and asthma symptoms, most pronouncedly in female participants. The results were consistent across several measures of asthma and in participants who reported never smoking. Associations with asthma outcomes could not be identified for snus use starting after age 15 years. Thus, our analyses suggest that using snus early in puberty may harm respiratory health more than if such use starts at a later age, independent of tobacco smoking.

### Study findings in context

#### Puberty: an age of vulnerability to tobacco products

To the best of our knowledge, this is the first study to address the important aspect of the age when starting snus and its association with asthma. A previous study of a Swedish population showed that current use of snus was associated with an increased risk of asthma and asthma-like symptoms,^6^ however, age of snus initiation was not available. On the other hand, there is available evidence regarding time of initiation of tobacco smoking, in which early onset has been shown to be important for respiratory disease. For instance, participants from a large British cohort who started smoking before the age 16 of years had a higher risk of obstructive airways disease in adult life compared with those who started smoking in adulthood^25^; similarly, Erbas *et al*, in an analysis of the ECRHS-I, found that compared with starting smoking tobacco at 16 years of age, an earlier age of initiation was associated with higher odds of asthma and respiratory symptoms in women.^14^ Puberty, thereby, seems to be a highly vulnerable life stage for the lungs; and nicotine from tobacco, whether smoked or placed in the mouth, may possibly be an important factor in the mechanism behind our findings on the higher risks of asthma symptoms when snus use initiates early.^9 11 13^

#### Stronger associations in women

Sex differences were notable in our analyses, especially our findings suggest that starting snus early was associated with more asthma symptoms in women. Our findings align with the existing evidence about sex-specific effects of smoking tobacco on respiratory health. In a previous study in Norway, young adolescent women who started smoking early (before age 16 years) reported more wheezing and nighttime dry cough in the past 12 months compared with men with similar tobacco-consumption habits.^26^ Patel *et al* also reported a stronger effect of smoking initiation in childhood on obstructive airway disease in adult women compared with men.^25^ Men and women exhibit differences in airway development, with men having larger small airway calibre while women experience a faster maturation of the respiratory system.^20^ Furthermore, hormonal distinctions between sexes may play a role in susceptibility of the lungs to external agents like tobacco,^27^ in addition to multiple other factors and mechanisms that may increase the risk to develop asthma (eg, genetics, previous allergies and occupation).^28^ Such sex differences could substantially contribute to explaining why early initiation of both tobacco smoking and snus use seems to influence respiratory health more strongly in women than in men.

### Strengths and limitations of this study

This study used data from a large multicentre population-based cohort, with a high prevalence of exposure to snus, ensuring robust statistical power to detect associations. We characterised the age of snus use initiation, considering covariates crucial to disentangle the relationship with asthma (eg, age of starting smoking and pack-years). Importantly, we addressed the impact of smoking tobacco through sensitivity analysis among participants who have never smoked, yielding consistent findings. The asthma definitions used were previously validated and each grasps different aspects of the disease; the asthma symptom score, in particular, has proved to be more specific,^22 29^ thus minimising potential misclassification biases (eg, diagnostic bias). This outcome, in addition, increases the statistical power of the analysis. The fact that the analyses using the different definitions show consistent results, strengthen the support for our hypothesis. Finally, we did not find any clear tendency for reverse causality of our observations, and we were able to analyse a clear temporal link between early initiation of snus use and respiratory outcomes, by including only outcomes being present in the last 12 months, therefore, enhancing the associations of early snus use.

The cross-sectional design, however, introduces some limitations, including misclassification of snus use and age of initiation due to self-reports, the stigma associated with its use, the stipulated legal age for snus consumption, legal restrictions on selling tobacco-containing snus in some of the countries where some study centres (eg, Aarhus, Reykjavik and Tartu) were located^30^ and the asthma status of the participants. In addition, recall bias about age of initiation may be another misclassification source, however, we expect it to be minor given the evidence supporting strong validity of self-reported smoking habits,^31^ which could potentially be similar to self-reports of snus use habits. Any misclassification, if present, is likely non-differential and may lead to underestimation of the tested associations. Other limitations include insufficient data on snus consumption amounts, which could impact dose–response associations, and potential residual confounding by factors like dual or intermittent use of snus and tobacco smoking. Finally, although the analyses included the main known covariates identified by DAG that could influence the associations, there may possibly be other factors that we have not accounted for, and unknown confounding cannot be ruled out.

### Implications

Our results show that snus use is popular in Northern Europe, even in countries without legal protection for snus trade such as Estonia, Denmark and Iceland.^30^ These findings add substantial knowledge to the existing by showing that early initiation of snus use is widespread and that there are indications it may increase the risk of asthma. This study highlights the susceptibility of puberty, during which consumption of snus could significantly influence respiratory health, particularly among young women. Additionally, it cannot be ruled out that other nicotine-containing products, such as e-cigarettes and nicotine pouches, may have similar effects as what we have found for snus, carrying important common public health implications. This information can guide future preventive interventions for high-burden diseases like asthma in settings where oral moist tobacco use is increasingly common. It also calls for attention from healthcare professionals and policymakers to address the alarming trend of early-life use of alternative tobacco and nicotine products.

## Conclusions

Our findings suggest an increased risk of asthma and asthma symptoms in persons starting using the nicotine-containing tobacco product ‘snus’ before or at 15 years of age, especially among never-smoking women. These results bring new evidence to the field of tobacco-related health effects, most likely also relevant for other nicotine-containing products and reinforce the growing knowledge of the plausible influence of snus on the respiratory system. Importantly, our study raises concerns about the possible detrimental health effect of early initiation of snus use and, consequently, about the safety and suitability of this product which is popular among the youngest.

## supplementary material

10.1136/bmjresp-2024-002401 online supplemental file 1

10.1136/bmjresp-2024-002401 online supplemental file 2

## Data Availability

Data are available on reasonable request.
