# Supplementary material for: Use of oral moist tobacco (snus) in puberty and its association with asthma in the population-based RHINESSA study
Source: BMJ Open Respir Res. 2024 Jul 22;11(1):e002401. doi: 10.1136/bmjresp-2024-002401 (PMC11268032; doi:10.1136/bmjresp-2024-002401)
Supplement: online supplemental file 2 [file bmjresp-11-1-s002.pdf]

Title: Use of oral moist tobacco (snus) in puberty and its association with asthma in the population-based RHINESSA study

SUPPLEMENTARY DATA

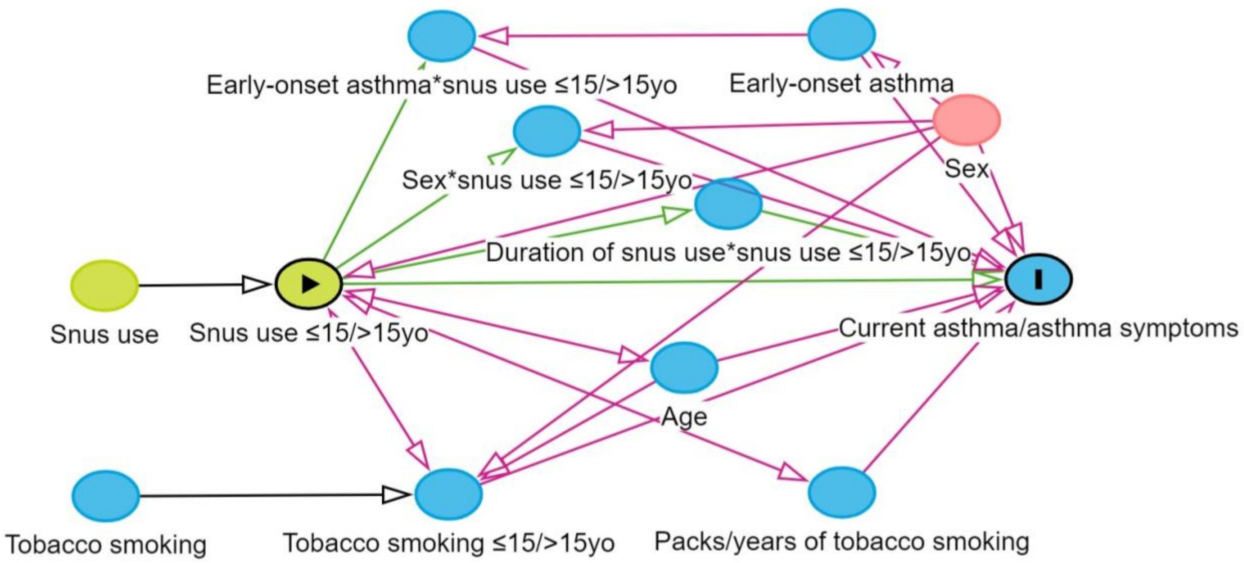

Figure S1. Direct Acyclic Graph (DAG) depicting the correlation between snus use ≤15 and >15 years old and current asthma/asthma symptoms, and the potential confounders (minimal sufficient adjustment set).

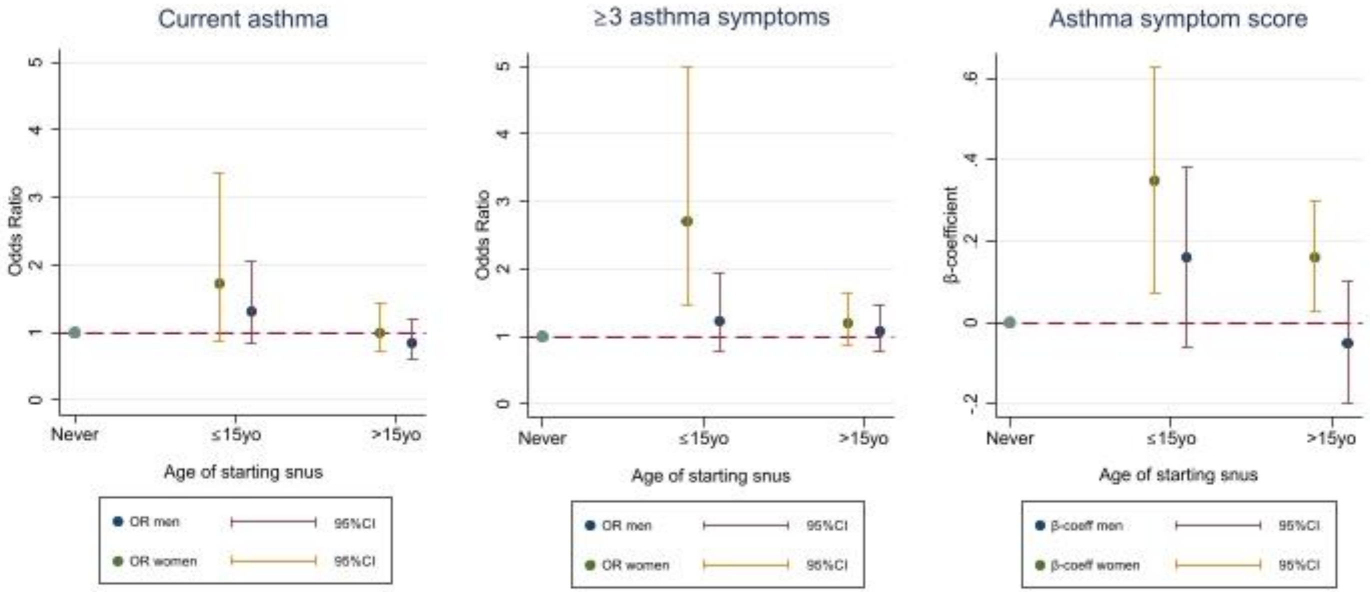

Abbreviations: OR= Odds Ratio; β=Beta coefficient; 95%CI= 95% Confidence Interval.

Figure S2. Plot of the estimates of the association of age of starting snus (never, ≤15 years old, >15 years old) with current asthma (left panel), ≥3 asthma symptoms (middle panel), and asthma symptom score (right panel) in all the participants.

Table S1. Current asthma and asthma symptoms according to age, study center and BMI, by sex.

	**Current asthma***		**≥3 asthma symptoms†**	
	Women	Men	Women	Men
**Age, median**	28.3	28.4	28.9	28.9
**Study centers, %**				
Aarhus (DK)	8.0	6.3	11.1	7.2
Bergen (NO)	10.8	6.7	14.8	9.6
Gothenburg (SE)	8.8	8.7	12.6	10.2
Umea (SE)	15.2	9.3	17.6	11.3
Uppsala (SE)	12.2	10.1	16.0	11.0
Reykjavik (IS)	13.1	5.8	18.1	9.6
Tartu (EE)	4.4	5.0	7.9	8.3
**BMI**				
< 18.5	5.1	3.3	5.5	5.7
≥ 18.5-24.9	9.7	6.9	12.7	8.8
≥ 25-29.9	11.9	8.2	17.5	9.9
≥ 30	19.8	7.5	26.4	14.4

Abbreviations: DK: Denmark; NO: Norway; SE: Sweden; IS: Iceland; EE: Estonia; BMI: Body Mass Index.

* Defined as: use of asthma medication in the last 12 months and/or asthma attacks in the last 12 months.

† Defined as: ≥ three positive answers to eight questions, based on a modified version of the definition provided by Pekkanen et al, 2005: Wheeze with breathlessness in the last 12 months; wheeze without cold in the last 12 months; woken by tightness in chest in the last 12 months; woken by attack of shortness of breath in the last 12 months; woken by night cough in the last 12 months; ever had asthma; asthma attack in the last 12 months; currently taking asthma medication.

Table S2. Asthma outcomes related to age of starting snus in those without early-onset asthma (before age 15 years), by sex.

	**Current asthma***		**≥ 3 asthma symptoms†**		**Asthma symptom score‡**	
No early-onset asthma	Women	Men	Women	Men	Women	Men
**Age of starting snus**	OR (95% CI)		OR (95% CI)		β (95% CI)	
Never	1 (ref)	1 (ref)	1	1	0	0
≤15 years old	1.49 (0.58-3.87)	1.76 (0.90-3.40)	1.93 (0.90-4.17)	1.34 (0.70-2.58)	0.11 (-0.25-0.47)	0.19 (-0.06-0.45)
>15 years old	0.82 (0.51-1.33)	1.07 (0.64-1.76)	1.14 (0.79-1.65)	1.25 (0.80-1.95)	0.15 (0.01-0.30)	-0.04 (-0.22-0.14)

Abbreviations: OR (95% CI) (Odds Ratio (95% Confidence Interval)); β (95% CI) (β coefficient (95% Confidence Interval)).

Model adjusted for: age, age of starting tobacco smoking (never, ≤15 years old, >15 years old) and packs/years of tobacco smoking.

* Defined as: use of asthma medication in the last 12 months and/or asthma attacks in the last 12 months. Data available for n=7795.

† Defined as: ≥ three positive answers to eight questions, based on a modified version of the definition provided by Pekkanen et al, 2005: Wheeze with breathlessness in the last 12 months; wheeze without cold in the last 12 months; woken by tightness in chest in the last 12 months; woken by attack of shortness of breath in the last 12 months; woken by night cough in the last 12 months; ever had asthma; asthma attack in the last 12 months; currently taking asthma medication. Data available for n=7795.

‡ Continuous scale based on the eight questions of the definition by Pekkanen et al, 2005. Data available for n=7702.

Table S3. Asthma with and without hay fever associated to age of initiation of use of oral moist tobacco, by sex.

	**Asthma with hay fever***		**Asthma without hay fever†**	
	Women	Men	Women	Men
**Age of starting snus**	OR (95% CI)		OR (95% CI)	
Never	1 (ref)	1 (ref)	1	1
≤15 years old	2.18 (1.03-4.65)	1.32 (0.78-2.24)	2.10 (1.10-4.10)	1.30 (0.70-2.33)
>15 years old	1.40 (0.96-2.04)	0.97 (0.67-1.41)	0.90 (0.62-1.31)	1.24 (0.82-1.85)

Abbreviations: OR (95% CI) (Odds Ratio (95% Confidence Interval)).

All models adjusted for: age, age of starting tobacco smoking (never, ≤15 years old, >15 years old) and packs/years of tobacco smoking. Data available for n=8328.

* Defined as: having current asthma and/or ≥3 asthma symptoms and ever having hay fever.

† Defined as: having current asthma and/or ≥3 asthma symptoms and never having hay fever.

Table S4. Ever use of snus and tobacco smoking associated with early-onset asthma (asthma ≤10 years old and between 11-15 years old), by sex.

	**Ever use of snus***		**Ever smoking tobacco†**	
	Women	Men	Women	Men
**Early-onset asthma‡**	aOR (95% CI)		aOR (95% CI)	
No	1 (ref)	1 (ref)	1	1
≤10 years old	1.33 (0.96-1.82)	0.88 (0.64-1.20)	1.09 (0.85-1.40)	0.84 (0.64-1.10)
11-15 years old	1.14 (0.77-1.69)	1.03 (0.61-1.74)	0.96 (0.70-1.30)	0.87 (0.54-1.40)

Abbreviations: aOR (95% CI) (Adjusted Odds Ratio (95% Confidence Interval)).

Model adjusted for age. Available data for n=9002.

* Defined as: never or ever using snus.

† Defined as: never or ever smoking tobacco.

‡ Defined as: age when first started with asthma symptoms (≤10 or 11-15 years old).
